# AI-Based Estimate of the Regional Effect of Orthokeratology Lenses on Tear Film Quality

**DOI:** 10.3390/bioengineering12101086

**Published:** 2025-10-06

**Authors:** Lo-Yu Wu, Wen-Pin Lin, Rowan Abass, Richard Wu, Arwa Fathy, Rami Alanazi, Jay Davies, Ahmed Abass

**Affiliations:** 1 Department of Optometry, Mackay Medical University, New Taipei 252, Taiwan; 2 Research and Development Centre, Brighten Optix Corporation, Taipei 111, Taiwan; 3 Department of Medicine, University of Cambridge, Cambridge CB2 0SP, UK; 4 College of Optometry, Pacific University, Forest Grove, OR 97116, USA; 5 Department of Engineering, University of Cambridge, Cambridge CB2 1PZ, UK; 6 Department of Materials, Design and Manufacturing Engineering, University of Liverpool, Liverpool L69 3GH, UK

**Keywords:** AI, cornea, tear film, tear film quality mapping, contact lens, orthokeratology, Ortho-K, spatial mapping, corneal topography, digital health

## Abstract

Purpose: To investigate regional changes in tear film quality associated with orthokeratology (Ortho-K) lens wear using high-resolution spatial mapping and to evaluate the potential of artificial intelligence (AI) models in anticipating these changes. Methods: This study analysed tear film quality in 92 Ortho-K wearers divided into three groups based on lens wear duration (10–29 days, 30–90 days, and ≥91 days). Placido-based topographer measurement was used to generate regional tear film maps before and after treatment. A custom MATLAB pipeline enabled regional comparisons and statistical mapping. A feedforward neural network was trained to forecast local tear film quality using spatial data. Results: Single-value global mean metrics showed minimal changes in tear film quality across groups. However, regional mean mapping revealed significant mid-peripheral and peripheral deterioration over time, particularly in nasal and temporal corneal zones. These changes were often overlooked by global averaging and remained invisible through tear film breakup time (TBUT) measurements. The AI model predicted spatial tear quality with high accuracy (R ≥ 0.9 in testing), capturing nuanced regional variations. Conclusions: The regional analysis uncovers subtle, clinically relevant tear film disruptions caused by Ortho-K lens wear, particularly in peripheral areas. These insights challenge the adequacy of traditional single-value global mean assessments. The AI model demonstrates the potential for non-invasive, predictive evaluation of tear stability, supporting more personalised and effective Ortho-K care.

## 1. Introduction

The tear film of the eye plays a critical role in maintaining ocular health and clear vision. It provides a smooth refractive surface while also protecting the eye from mechanical stress and microbial invasion [1,2]. When the tear film is compromised, whether through chemical imbalance or reduced production, patients may develop dry eye disease, blurred vision, and even corneal or conjunctival infections [3,4,5]. The most widely used clinical test of tear film stability is tear film breakup time (TBUT), which measures the interval between a blink and the first appearance of tear film disruption [5].

Although TBUT offers a simple estimate of tear film evaporation, it does not fully capture the complexity of tear film behaviour. A single time value cannot describe regional variations across the corneal surface, and the measurement itself is influenced by blinking patterns, examiner interpretation, and environmental factors such as humidity and airflow [6,7]. Averaging techniques, while intended to reduce variability, further mask meaningful local fluctuations. Factors such as blinking behaviour, eyelid anatomy, and contact lens wear often create regional differences that are lost when relying only on global metrics [8,9]. As a result, subtle regional disruptions, sometimes early signs of dry eye disease, may go unnoticed [10].

Previous studies of orthokeratology (Ortho-K) have largely relied on TBUT or subjective reports of dryness to evaluate tear film changes [11,12]. While TBUT remains practical in clinics, its subjectivity, low repeatability, and lack of spatial resolution limit its usefulness. A single scalar value may obscure local tear film instability, particularly in the mid-peripheral and peripheral cornea, areas most affected by the mechanical forces of Ortho-K lens wear [13,14].

Orthokeratology is a widely used, non-surgical technique in which rigid contact lenses are worn overnight to temporarily reshape the cornea [15]. It is especially popular for myopia control in children and adolescents [16,17]. However, by altering the corneal surface profile, Ortho-K lenses inevitably interact with the tear film, introducing localised pressure and surface irregularities that may disrupt tear stability [9,18].

The literature is not in agreement about the impact of Ortho-K on tear film stability, and conflicting findings may partly reflect the reliance on single-value or averaged assessments [19,20]. To address this gap, the present study applies Placido-based tear film mapping to generate a spatially resolved view of tear film quality before and after Ortho-K treatment. Regional analysis offers a clear advantage over traditional single-value measures such as global averages or TBUT, as it can reveal local tear film instability in zones most affected by lens pressure and decentration. Detecting these subtle changes has direct clinical value, as it can guide follow-up care, support timely adjustments to lens fitting, such as using lubricating drops, and help reduce the risk of ocular surface complications.

The study also introduces a first-of-its-kind AI-based model capable of predicting complete tear quality maps rather than single averaged values. This AI-based tool has strong potential for clinical practice, where it could be used to anticipate timelines of increased risk, support more tailored Ortho-K management, and ultimately enhance wearer comfort and long-term ocular safety.

## 2. Materials and Methods

Participants were divided into three groups according to the duration of Ortho-K lens wear: 10–29 days (n = 13), 30–90 days (n = 28), and 91 days or more (n = 51). These ranges were selected to represent the early adjustment phase, an intermediate stage, and established long-term wear. The distribution of participants across groups was not uniform, reflecting typical clinical practice in which fewer patients are reviewed at the earliest stages, while the majority progress to sustained lens wear. Although the smaller sample size in the early-wear group may limit the detection of subtle short-term changes, its inclusion was considered important to capture initial tear film responses. By contrast, the larger intermediate and long-term groups enhance the robustness of the findings and allow for more reliable interpretation of progressive and cumulative effects. The clinical data collection was approved by the Institutional Review Board of Taipei Medical University, Taiwan, and conducted in accordance with the Declaration of Helsinki.

### 2.1. Corneal Topography and Tear Film Quality Analysis

A custom-built MATLAB 2025b (MathWorks Inc., Natick, MA, USA) pipeline was developed to process and visualise spatial maps from the Medmont E300 to evaluate the impact of Ortho-K on corneal shape and tear film quality. For each subject, right eye (oculus dexter) and left eye (oculus sinister) (OD/OS), raw pre- and post-treatment data with tear film quality maps were exported as Medmont export file (MXF) collections, loaded via a bespoke MATLAB coded reader, and parsed into analysis matrices.

The pipeline segmented and saved both modalities at both time points and produced a standard six-map layout to support pointwise interpretation: pre- and post-tangential curvature, a curvature difference map (post–pre), pre- and post-tear film quality, and a tear film quality difference map (post–pre). Maps were quality-checked, low-pass filtered where appropriate, and resampled onto a standard corneal grid. Regional tear film quality factors were extracted at 121 predefined pivot locations, yielding a supervised dataset with wear duration (days) as the single input and 121 regional outputs. The selection of the distribution of pivot locations is detailed in Appendix A.

Candidate feedforward neural networks were screened automatically via a MATLAB-coded grid search (see Section 2.3 for more details). The parsimonious 1, (5, 8), 121 architecture achieved the lowest validation error with good generalisation and was therefore selected. The final model was trained with the Levenberg–Marquardt optimiser using mean-squared error, with a 70%, 15%, 15% train, validation, test split, and default “mapminmax” preprocessing. The network predicts a full regional tear film map for any given wear time, which is interpolated for visualisation. Pointwise Wilcoxon signed-rank tests were applied at each map location, with Benjamini–Hochberg FDR correction, to identify significant pre- versus post-treatment changes.

**Figure 1:**
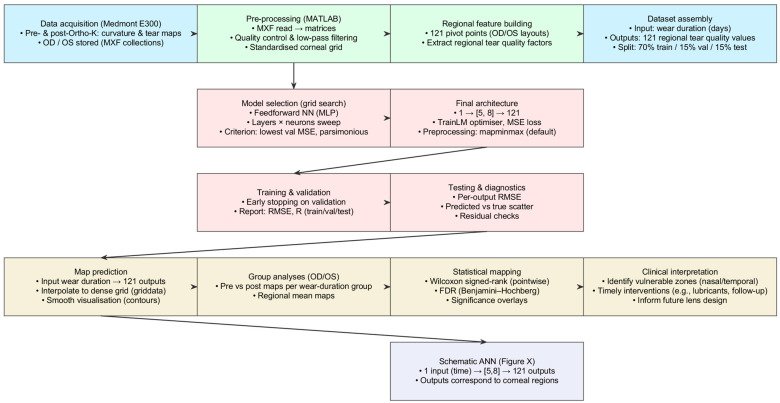
Workflow from data to AI prediction and clinically oriented spatial analysis.

All spatial data (curvature, tear film quality, and calculated centration matrices) were saved for subsequent statistical analysis. Cases with inadequate image quality were excluded, defined as low contrast, excessive blinking artefacts, or incomplete coverage of the central 3.5 mm (Figure 1).

### 2.2. Group-Wise Evaluation of Tear Film Quality over Time

A group-based analysis was conducted using a custom-built MATLAB 2025b script to assess how tear film quality changes across different Ortho-K wear durations. The script loaded regional tear film quality maps for both pre- and post-treatment sessions, analysing right and left eyes separately throughout the study. This separation was maintained for all group-level averaging, visualisation, and statistical analyses to account for anatomical and tear film symmetry differences between eyes to avoid the influence of fellow eyes’ natural correlation, and to consider reported differences among fellow eyes [21,22,23].

Participants were ranked into three groups based on their Ortho-K lens wear duration: Group 1 (10–29 days), Group 2 (30–90 days), and Group 3 (91 days or more). For each group, mean pre- and post-treatment tear quality maps were computed by averaging across all eyes in the respective time range. The averaged maps were displayed using polar-contoured visualisations over a fixed corneal coordinate grid to highlight regional variations. A custom colour map was applied to enhance interpretability, and graphical overlays were added to indicate centration and spatial scales.

Tear film quality values were extracted from concentric polar pattern rings at predefined angular intervals using piecewise cubic interpolation to quantify changes [24]. This allowed for a structured comparison of regional tear stability over time. Regional mean tear quality values were calculated for each eye and group and used for visual plotting and statistical analysis.

To visualise group-level differences, violin plots were generated to illustrate the distribution of tear quality values in each group, highlighting central tendencies (mean, median, and mode) and statistical comparisons between groups. Violin plots were chosen because they show the average values and the full spread of the data. Unlike simple bar charts, they reveal the shape of the distribution, whether the data are tightly clustered, spread out, or have multiple peaks. This makes them especially valuable for comparing group differences and showing essential details. In these violin plots, the mean represents the global average value, the median is the global middle value, and the mode is the global most common value.

### 2.3. Artificial Intelligence Prediction Approach

This study employed an artificial intelligence (AI)-based method using a feedforward neural network (FNN) to pattern and predict the spatial distribution of tear film quality. Neural networks are machine learning models mimicking biological neural systems. They are particularly effective for modelling non-linear, complex relationships, making them ideal for clinical prediction tasks in ophthalmology and beyond [25,26].

A multilayer perceptron (MLP) architecture was adopted. The MLP is an FNN that consists of an input layer connected to one or more hidden layers, and delivers the outcome to an output layer, with full connectivity between neurons across layers. The model was trained using supervised learning to minimise mean-squared error (MSE); RMSE is reported for interpretability between predicted and actual regional tear quality values [27].

Each network output corresponds to a spatial pivot point across the corneal surface, enabling localised predictions. This spatially resolved approach allowed the model to detect subtle regional changes that may be overlooked by conventional tear film assessments based on global averaging [28]. The model’s strength lies in its ability to map these variations using a single-shot input, reflecting a finer level of diagnostic detail.

The FNN was utilised to predict regional tear film quality, with its architecture optimised using a systematic grid search. This approach exhaustively tested combinations of two hyperparameters: the number of hidden layers (ranging from 1 to 10) and the number of neurons per layer (8, 16, 32, and 64). These values were selected to balance computational efficiency with sufficient model complexity, covering a range of shallow to deeper architectures commonly used in biomedical modelling. In total, 40 unique FNN configurations were evaluated. Each model was trained on 70% of the data, with 15% used for validation and 15% for testing, a widely accepted split that ensures robust generalisation while preserving enough data for performance evaluation. The optimal network was selected based on the lowest validation RMSE, helping mitigate overfitting. The final model performance was assessed using RMSE and correlation analyses, confirming its reliability in capturing spatial tear film variability.

**Figure 2:**
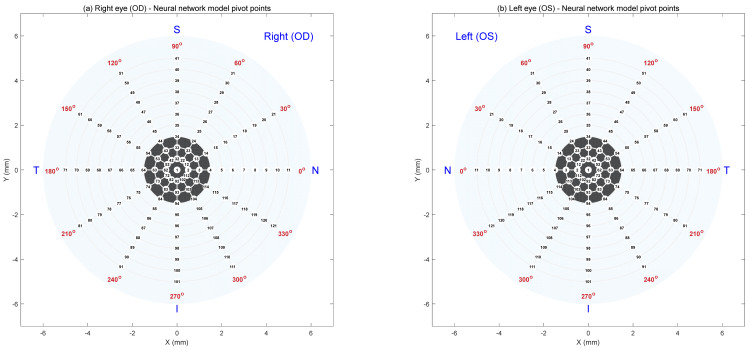
Schematic showing the (**a**) right and (**b**) left eye pivot points used to construct the neural network model. “N” stands for the nasal part of the eye (towards the nose), “T” for temporal (towards the temples), “S” for superior (the upper part), and “I” for inferior (the lower part).

In order to facilitate the regional mapping prediction, the AI FNN model was built using a grid of pivot points distributed across the corneal surface, following [29], to enable spatially detailed predictions of tear film quality. These points aligned with physiologically relevant regions, allowing the FNN to detect localised variations rather than relying solely on global averages. Separate grid point orders were created for the right and left eyes to account for their natural anatomical differences [23]. Figure 2 illustrates this layout, where subplot (a) displays the pivot point arrangement for the right eye and subplot (b) for the left. Each map contains 121 evenly spaced points arranged radially from the centre of the cornea to the periphery. These pivot points served as the output locations for the AI model, enabling precise, region-specific predictions of tear film quality across the ocular surface.

**Figure 3:**
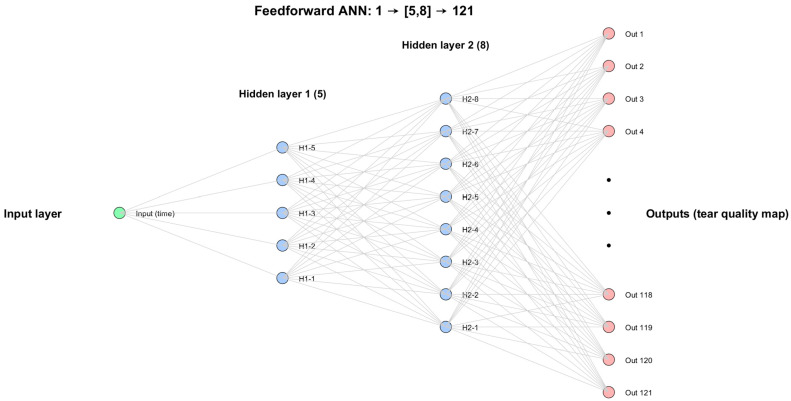
The architecture of the FNN used to predict spatial tear film quality maps from orthokeratology wear time. The model consists of one input neuron (wear duration), two hidden layers with 5 and 8 neurons, respectively, and 121 outputs, building the tear map.

After testing several configurations automatically through a custom-built MATLAB 2025b code, the final architecture was selected as the most efficient compromise between accuracy and simplicity. It consisted of a single input neuron representing wear time, two hidden layers with 5 and 8 neurons, respectively, and 121 output neurons corresponding to tear film quality values at predefined corneal locations. This configuration was identified through grid search experiments, which compared models of varying depth and size. The (5, 8) structure consistently produced the lowest validation error while avoiding overfitting, making it the most reliable model for capturing the spatial patterns of tear film change. The compressed schematic of the network is presented in Figure 3.

### 2.4. Statistical Analysis

For statistical comparison, Wilcoxon signed-rank tests were conducted at each map location to detect spatially significant differences in tear film quality before and after treatment [30,31]. This nonparametric test was selected because it does not assume a normal distribution and is therefore appropriate for modest sample sizes and paired, non-Gaussian data. Statistical analyses were performed using custom-built scripts in MATLAB 2025b. Significance *p*-values below 0.05 were considered significant, and results were displayed as contours of overlaid significance on the maps.

To address the increased risk of false positives associated with multiple comparisons across the corneal surface, FDR adjustment was applied using the Benjamini–Hochberg procedure at a significance level of 0.05. Adjusted *p*-values were incorporated into regional significance maps, which were overlaid onto the mean tear film quality maps for right and left eyes separately.

Between-group differences in tear quality (based on lens wear duration) were assessed using group-wise averaging and violin plot visualisations. Pairwise comparisons were performed with nonparametric rank-sum tests, chosen for their robustness in settings with unequal and relatively small group sizes. Given non-Gaussian distributions and potential outliers, associations between tear quality and clinical parameters (e.g., wear time, horizontal visible iris diameter) were assessed using Spearman’s rank correlation R. Two-sided 95% confidence intervals were obtained via bootstrap resampling (10,000 iterations), and statistical significance was set at *p* < 0.05.

Although the smallest group (10–29 days, n = 13) limits statistical power, the combination of nonparametric methods and FDR correction provides a conservative and reliable approach. The larger intermediate and long-term groups (30–90 days, n = 28; ≥91 days, n = 51) strengthen the robustness of group comparisons, while the regional mapping approach increases sensitivity to subtle, localised changes that may otherwise be masked in global analyses.

## 3. Results

### 3.1. Typical Global Mean Analysis

Using typical global mean analysis, Table 1 shows the Spearman rank correlation (R) between the global mean tear film quality factor and wear time, as the lenses were worn in the right and left eyes. It should be noticed that the lower the global mean tear quality factor, the better the tear film quality; therefore, a positive correlation indicates a decline in tear quality. For Group 1, the right eye correlation was 0.02; the left eye was slightly higher, at 0.28. In Group 2, the correlations for both eyes were more similar, at 0.08 for the right eyes and 0.07 for the left eyes. Finally, Group 3 showed the highest correlation values, with 0.26 for the right eyes and 0.43 for the left eyes, suggesting a stronger relationship between wear time and global mean tear quality factor as the duration of Ortho-K use increases.

**Figure 4:**
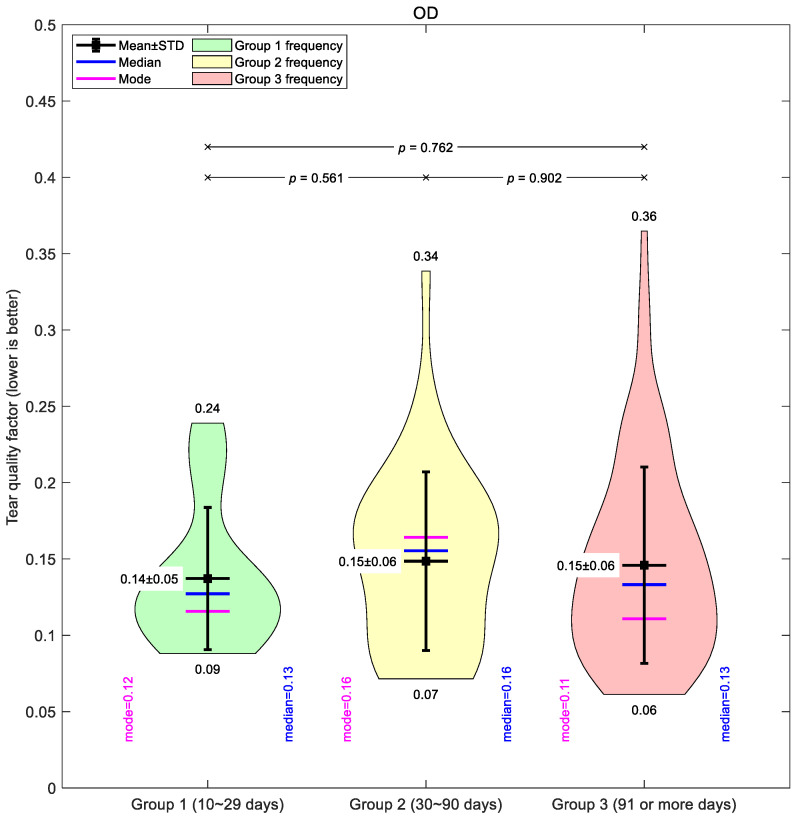
Right eyes tear film quality factor typical averaging for the three investigated Ortho-K wear groups: Group 1 (10~29 days), Group 2 (30~90 days), and Group 3 (91 or more days).

Figure 4 illustrates the global mean tear quality factor for the right eyes. Across all groups, the averages were similar, with only subtle differences between short-, medium-, and long-term wearers. Group 1 showed the lowest mean value (0.14 ± 0.05), suggesting slightly better tear quality in the early wear stage. Groups 2 and 3 both averaged 0.15 ± 0.06, though Group 3 displayed the widest spread of values (0.06–0.36), indicating greater variability in longer-term wearers.

**Figure 5:**
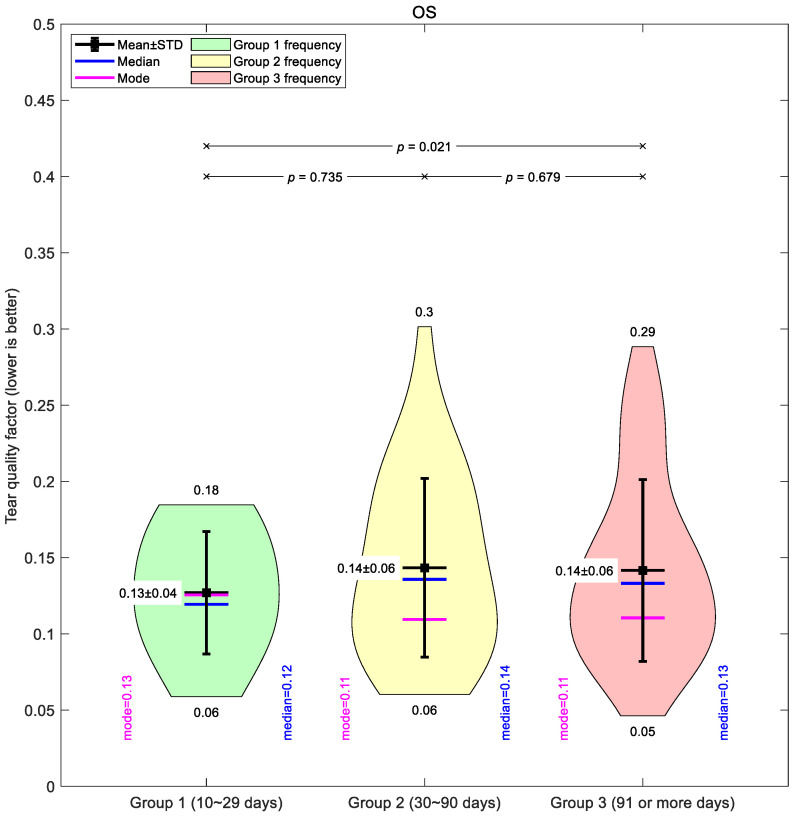
Left eye tear film quality factor typical averaging for the three investigated Ortho-K wear groups: Group 1 (10~29 days), Group 2 (30~90 days), and Group 3 (91 or more days).

A comparable pattern was seen in the left eyes (Figure 5). Group 1 had a mean of 0.13 ± 0.04, again the lowest of the three. Groups 2 and 3 averaged 0.14 ± 0.06, with Group 3 showing a similarly broad range (0.05–0.29). Across both eyes, the medians and modes were closely aligned with the means, supporting the overall constancy of the global average values that may not show regional variations in depth.

Taken together, the global mean data suggest only modest changes in tear film quality with increased lens wear, and the differences between groups were small compared to the variability within each group.

### 3.2. Regional Mean Map

The regional mean maps revealed distinct spatial patterns in tear film quality that varied with lens wear duration. Compared to pre-treatment conditions, post-Ortho-K maps consistently showed peripheral deterioration and altered symmetry, particularly in mid and long-term wearers. Central tear quality was generally maintained in the early stages but became more localised or diminished over time. Results summarise these findings for right and left eyes, highlighting regional disruptions that were not evident through the global mean tear factor alone, especially on the nasal and temporal sides. These results underscore the importance of spatial analysis in detecting early signs of tear film instability during Ortho-K treatment. Detailed observations for each group are described in the following text.

**Figure 6:**
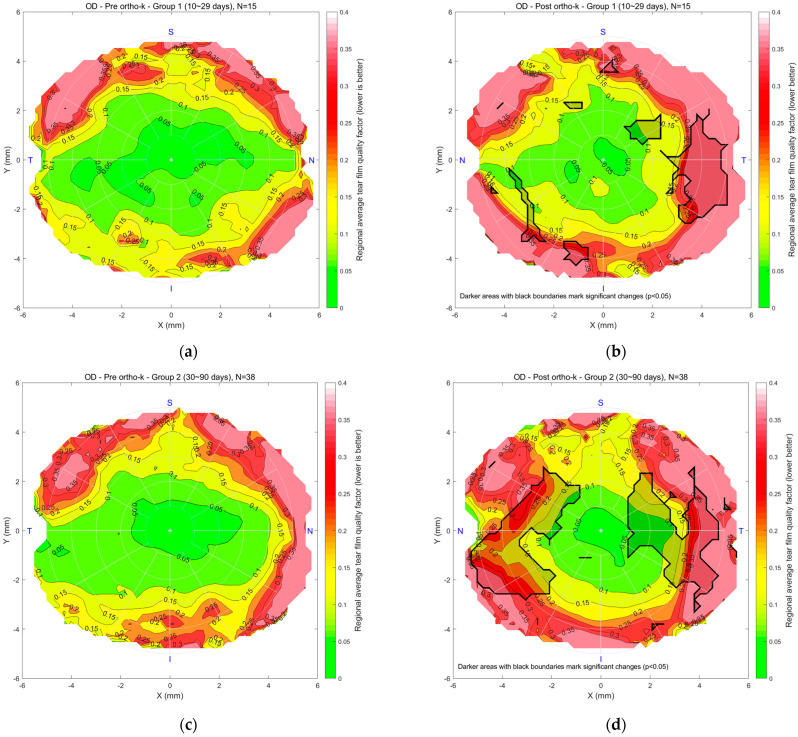

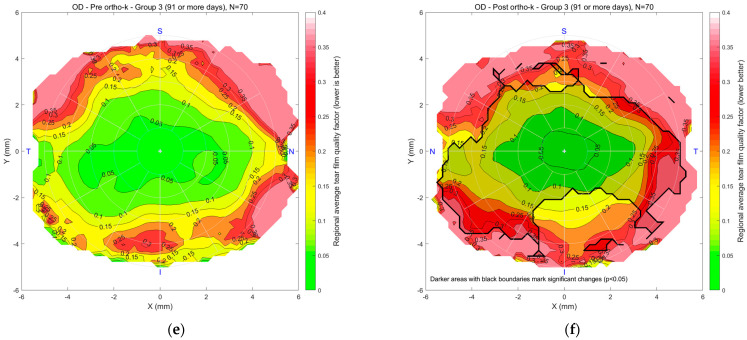
Right eyes regional mean analysis for Group 1 (10~29 days), (**a**) pre-Ortho-K and (**b**) post-Ortho-K, Group 2 (30~90 days), (**c**) pre-Ortho-K and (**d**) post-Ortho-K, Group 3 (91 or more days), (**e**) pre-Ortho-K and (**f**) post-Ortho-K. Notable patterns include stable central quality in early wearers and progressive peripheral deterioration in long-term users.

Figure 6 illustrates the regional mean tear film quality map for the right eyes in Groups 1, 2, and 3, comparing pre- and post-Ortho-K conditions. In maps (a) and (b) for Group 1, the colour scale indicates tear quality, with green representing better and red indicating poorer tear quality. “N” stands for nasal, the side of the eye closest to the nose. “T” means temporal, towards the temples. “S” is superior, or the top of the eye, and “I” is inferior, the bottom part. The pre-Ortho-K map shows a central area of good tear quality, with degradation toward the periphery. After 10–29 days of Ortho-K lens wear, the post-Ortho-K map reveals a slight improvement in the central region regarding the rotational symmetry of the tear quality. However, peripheral tear quality worsened beyond 4 mm diameter with a significantly worsened spot on the temporal side. In Group 2, maps (c) and (d) show a similar trend, with a small 2 mm diameter stable central region but a reduction in tear quality in peripheral areas after 30–90 days of Ortho-K use. Worsening spots with significant deterioration were noticed on both the nasal and temporal sides. For Group 3, maps (e) and (f) compare pre- and post-Ortho-K conditions after 91 or more days of lens wear. The post-Ortho-K map indicates a shrinking central area of good tear quality but noticeable improvement in surrounding regions, particularly closer to the centre, despite poorer quality in the peripheral areas. Changes were significant on the nasal, central, and temporal sides.

**Figure 7:**
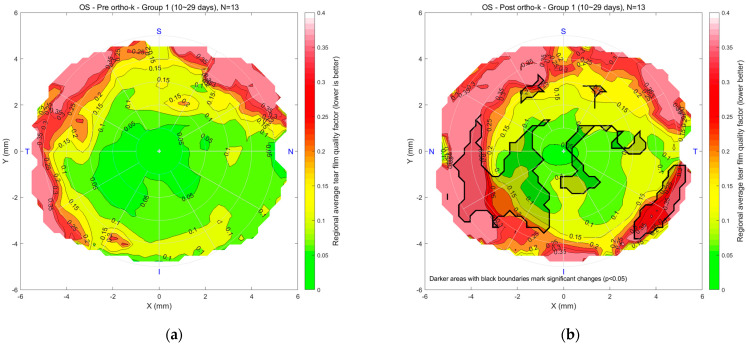

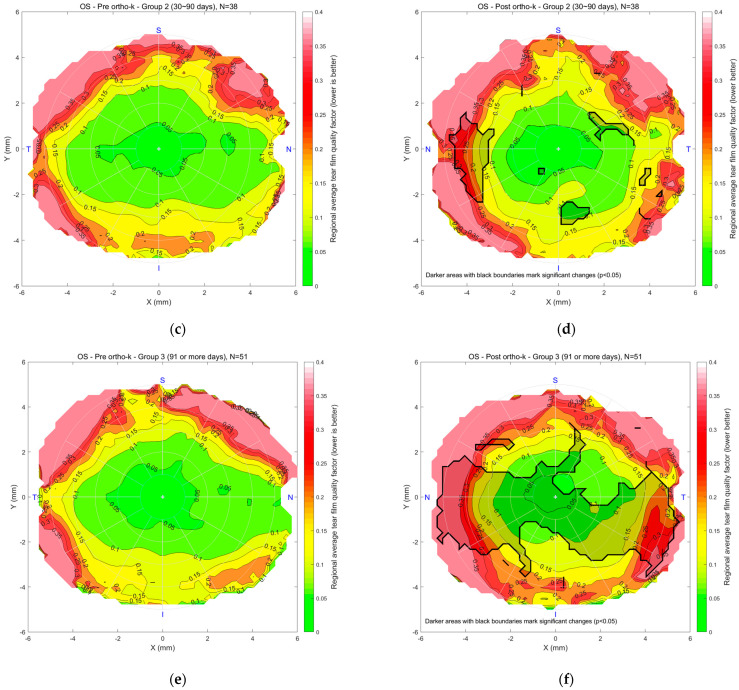
Left eyes regional mean analysis for Group 1 (10~29 days), (**a**) pre-Ortho-K and (**b**) post-Ortho-K, Group 2 (30~90 days), (**c**) pre-Ortho-K and (**d**) post-Ortho-K, Group 3 (91 or more days), (**e**) pre-Ortho-K and (**f**) post-Ortho-K.

Figure 7 presents tear quality data for the left eyes, with a similar colour scale from green to red. For Group 1 (n = 13), maps (a) and (b) compare pre- and post-Ortho-K conditions after 10–29 days of lens wear. The central region’s good tear quality area shrank, there was some mid-peripheral deterioration, and the outer periphery showed poorer tear quality. A significant deterioration spot was noticed on the nasal side. In Group 2 (n = 28), maps (c) and (d) compare pre- and post-Ortho-K conditions after 30–90 days of wear, showing more rotational symmetry in mid-peripheral regions, though peripheral areas remain poorer. In Group 3 (n = 51), maps (e) and (f) compare pre- and post-Ortho-K conditions after 91 or more days of lens wear. The post-Ortho-K map reveals similar central tear quality but significant improvement in mid-peripheral regions. At the same time, some peripheral areas still exhibit poorer tear quality, although overall tear distribution appears improved in terms of rotational symmetry.

**Figure 8:**
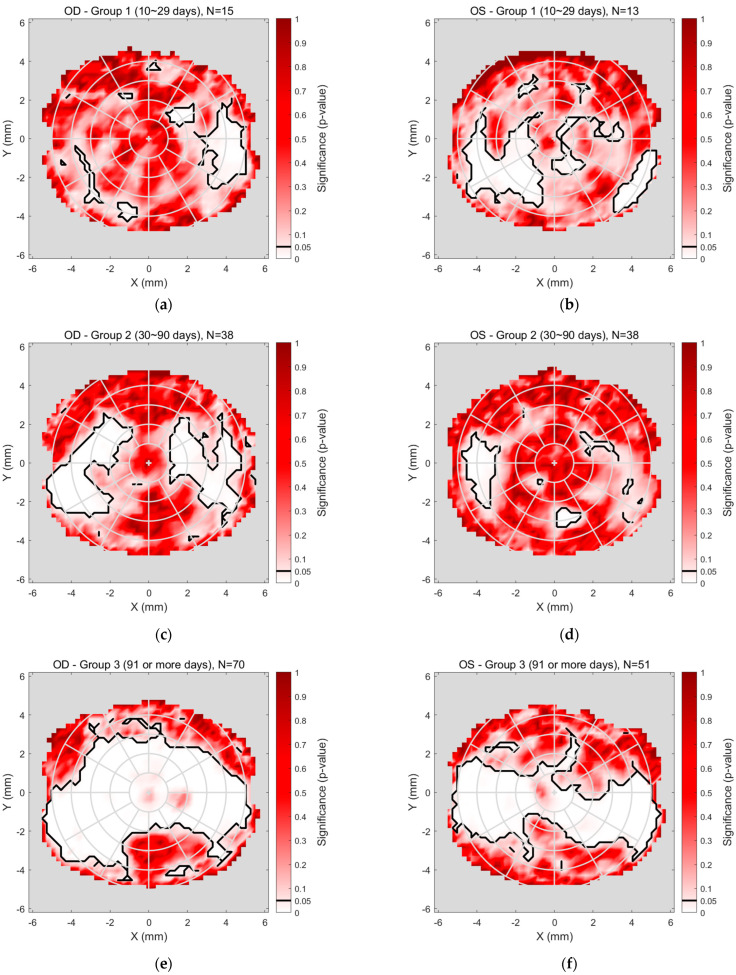
Significance mapping on regional analyses comparing pre- and post-Ortho-K wear for Group 1 (10~29 days), (**a**) right eyes and (**b**) left eye, Group 2 (30~90 days), (**c**) right eyes and (**d**) left eye, Group 3 (91 or more days), (**e**) right eyes and (**f**) left eye.

The detailed regional significance analyses, superimposed in Figure 6 and Figure 7, are presented more clearly in Figure 8, which illustrates the regional significance mapping of tear film quality, comparing pre- and post-Ortho-K lens wear across three study groups. The maps display areas of significant change in tear film quality for both the right and left eyes, with colour-coded regions representing the magnitude of significance *p*-value.

For the right eyes (Figure 8a,c,e), Group 1 (Figure 8a), representing the early stage of Ortho-K wear (10–29 days), showed minimal changes in tear film quality. Some significant differences were noted in the mid-peripheral areas, while the central and peripheral regions remained unchanged significantly. In Group 2 (Figure 8c), covering 30–90 days of Ortho-K lens wear, more pronounced changes were observed, particularly in the mid-peripheral regions, where significant changes in tear quality occurred, while the central area demonstrated no significant change. Group 3 (Figure 8e), reflecting extended Ortho-K wear (91 days or more), revealed a more complex pattern, with the central and peripheral areas displaying notable changes.

For the left eyes (Figure 8b,d,f), Group 1 (Figure 8b) showed some regional changes, including slight mid-peripheral changes and a significant change toward the nasal-inferior corner. In Group 2 (Figure 8d), no significant changes were observed in the mid-peripheral regions, with the outer periphery showing minimal changes in tear film quality, except for a notable change on the nasal side. Group 3 (Figure 8f) almost mirrored the findings for the right eye, as the quality of the nasal, central, and temporal tear film changed significantly. At the same time, the upper and inner peripheral regions were unchanged significantly.

### 3.3. AI-Driven Modelling

This section evaluates how well the AI model predicts tear quality in both right and left eyes during training and testing. The analysis centres on two key metrics, Spearman’s rank correlation R and RMSE, to measure the accuracy and reliability of the predictions.

**Figure 9:**
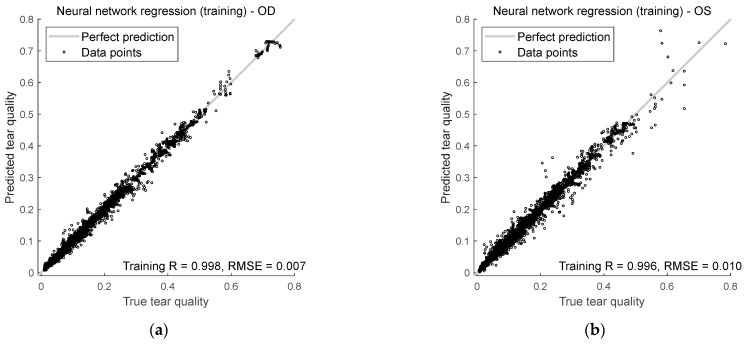

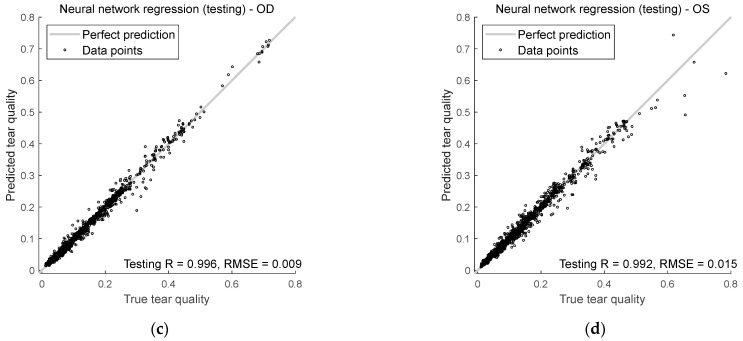
Neural network regression performance in (**a**) right eye training, (**b**) left eye training, (**c**) right eye testing, and (**d**) left eye testing.

Figure 9 shows how well the model predicts tear quality for both right and left eyes during training and testing. It uses two key measurements: Spearman’s R and RMSE.

For the right eyes, Spearman’s rank correlation during training was 0.998, indicating that the model’s predictions aligned well with the true tear quality values. The correlation dropped slightly in testing to 0.996, but was still durable, showing that the model continues to do well even with unseen input data. The RMSE for training and testing was lower than 0.01, suggesting that the model was precise when predicting the right eye’s tear quality.

For the left eyes, Spearman’s rank correlation coefficients were 0.996 during training and 0.992 in testing. While slightly lower than for the right eyes, these numbers still show a strong relationship between the predicted and actual values. The RMSE for left eyes was less than 0.02 in both the training and testing phases, meaning the model’s predictions for left eyes were slightly less accurate than right eyes but still very close to the actual values.

## 4. Discussion

This study provides new insights into how Ortho-K lenses affect tear film quality over time, focusing on regional variations across the corneal surface. The findings highlight the limitations of conventional global tear quality metrics and demonstrate the advantages of regional analyses for uncovering clinically relevant patterns.

Wilcoxon signed-rank tests were conducted pointwise at each spatial location of the tear quality maps to evaluate changes before and after treatment. This nonparametric approach was chosen because regional tear film values did not follow a Gaussian distribution, and the design involved repeated measures. Although the early-wear group was relatively small (n = 13), the use of nonparametric methods and FDR correction provided a conservative and reliable framework for analysis. The larger intermediate and long-term groups (n = 28 and n = 51, respectively) strengthened the robustness of the findings, while the regional mapping approach enhanced sensitivity to subtle, localised changes that global averages would likely obscure.

Traditional global mean methods offered limited insight in this context. Averaged tear quality factors remained relatively stable across groups, with only modest changes in mean values and almost no significant differences observed between wear durations. This approach failed to reveal where, and to what extent, the tear film was affected by Ortho-K wear. For example, in long-term wearers (Group 3), global metrics suggested only slightly worse tear quality, masking the regional disruptions identified through spatial mapping.

By contrast, regional mean analysis uncovered consistent and spatially distinct alterations, particularly in the mid-peripheral and peripheral regions. While the central tear film remained relatively preserved during early wear, peripheral degradation became increasingly apparent over time. In Groups 2 and 3, nasal and temporal sectors showed statistically significant deterioration. These patterns were confirmed through FDR-corrected spatial significance maps, highlighting the mechanical influence of lens design and potential lens decentration.

Interestingly, long-term wearers displayed improvements in mid-peripheral rotational symmetry alongside localised worsening, suggesting an adaptive reshaping of the ocular surface coupled with focal tear instability. These findings reinforce the view that tear film behaviour under Ortho-K is non-uniform and evolves with duration of use.

This study also introduces a novel way of presenting statistical significance through spatial maps, rather than relying on single-value outputs. By visualising where changes are statistically meaningful, clinicians gain a more intuitive and actionable understanding of lens-related effects. This regional mapping approach provides a powerful alternative to traditional scalar *p*-values, supporting more precise and location-specific decision-making in Ortho-K follow-up care.

The AI model successfully predicted regional tear film quality with high accuracy and strong correlation values, even on unseen test data. Its ability to capture non-linear spatial interactions suggests strong potential for clinical application, such as flagging early signs of regional instability or predicting post-treatment outcomes from pre-fitting topography. As AI-based tools continue to evolve in ophthalmology, the model demonstrated here makes a compelling case for their role in non-invasive, real-time evaluation [28,32,33].

These findings suggest that single-value tear film assessments are insufficient when managing Ortho-K patients. Spatially resolved data provide a clearer picture of ocular surface health and may better inform follow-up strategies, particularly when patients report discomfort or fluctuating vision. Incorporating regional analysis into routine assessments could enable more tailored lens designs and treatment plans.

Traditional assessments such as TBUT remain popular due to their simplicity, yet TBUT is limited by subjectivity, low repeatability, and the absence of spatial resolution. It produces only a single scalar value, overlooking subtle, region-specific disruptions, particularly in mid-peripheral and peripheral zones that are most affected by Ortho-K. In contrast, the regional mapping and AI-driven prediction presented in this study offer a more comprehensive and objective evaluation of tear film behaviour. By detecting patterns that TBUT cannot, this approach paves the way for more data-driven clinical care.

A limitation of this study is the absence of a non-Ortho-K control group, which would have clarified the extent to which the observed changes were treatment-specific rather than due to normal inter-visit variation. Future work should also examine how regional tear film disruptions relate to patient-reported outcomes such as visual clarity, ocular discomfort, and dryness symptoms, to strengthen the link between objective measures and clinical experience. Longitudinal studies extending beyond 120 days would be valuable to capture longer-term adaptation or degradation trends.

## 5. Conclusions

This study demonstrates that regionally resolved analysis of tear film quality yields clinically meaningful insights that are obscured by conventional global metrics, such as TBUT or mean averaging. Although global indices appeared largely stable across different durations of Ortho-K wear, spatial mapping revealed consistent and progressive deterioration in the mid-peripheral and peripheral cornea, particularly within the nasal and temporal sectors. These localised disruptions represent early indicators of ocular surface compromise that would likely be overlooked in routine assessments.

The clinical implications of the study are tangible. Longitudinal regional tear film mapping allows practitioners to detect instability at a pre-symptomatic stage, enabling timely interventions, such as recommending lubricating drops, adjusting follow-up schedules, or refining lens parameters, that may help prevent discomfort, visual fluctuation, or dropout. Beyond immediate management, these findings may also inform future Ortho-K lens design, supporting the development of geometries that better preserve tear film stability in vulnerable regions. Furthermore, introducing an AI-based model capable of predicting complete tear quality maps suggests a future pathway towards predictive chairside tools, allowing clinicians to anticipate periods of increased risk and manage Ortho-K wear with greater precision.

While further clinical evaluations in larger, more diverse cohorts and lens designs are warranted, this study underscores the importance of moving beyond single-value assessments towards spatially resolved, data-driven approaches. By revealing subtle yet clinically significant patterns of tear film disruption and offering immediate and long-term strategies for their mitigation, this work lays the foundation for safer, more personalised, and ultimately more successful Ortho-K treatment.

## Figures and Tables

**Table 1:** Rank correlation of tear quality with wear duration (lower = better quality).

Ortho-K Wearing Time	N	Right Eye (OD) Global Mean Tear Quality (Spearman R) vs. Wear Time (Lower Is Better)	Left Eye (OS) Global Mean Tear Quality (Spearman R) vs. Wear Time (Lower Is Better)
Group 1 (10~29 days)	13	0.02	0.28
Group 2 (30~90 days)	28	0.08	0.07
Group 3 (91 or more days)	51	0.26	0.43

**Figure A1:**
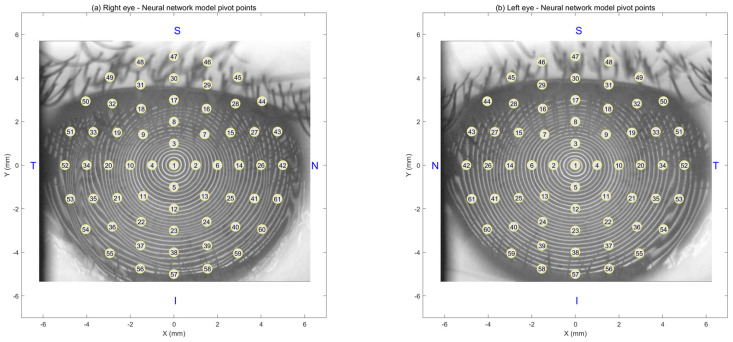
Concentric ring pattern, (**a**) right eye, (**b**) left eye.

**Figure A2:**
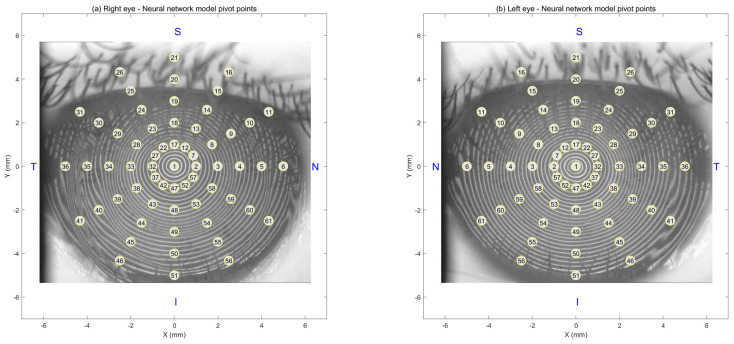
Concentric polar pattern, (**a**) right eye, (**b**) left eye.

**Figure A3:**
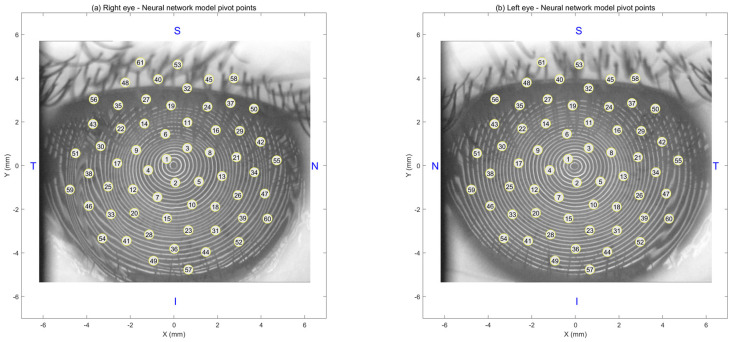
Sunflower spiral pattern, (**a**) right eye, (**b**) left eye.

## Data Availability

The datasets generated and analysed during the current study are available from the corresponding author on reasonable request. They are not publicly available due to potential future commercialisation.

## Abbreviations

The following abbreviations are used in this manuscript:

AI	Artificial Intelligence
FNN	Feedforward Neural Network
MLP	Multilayer Perceptron
MXF	Medmont export file
OD	Right eye (oculus dexter)
Ortho-K	Orthokeratology
OS	Left eye (oculus sinister)
R	Spearman’s rank correlation coefficient
RMSE	Root Mean Squared Error
TBUT	Tear Film Breakup Time

## Appendix A

This section provides further insight into selecting pivot locations across the corneal surface to construct a novel AI-based model for predicting tear film quality over time with Ortho-K lens wear. The placement of these pivots is essential, as they serve as representative points from which the overall tear quality map can be reconstructed.

To investigate this, three distinct pivot location patterns were evaluated: the concentric ring pattern (Figure A1), the concentric polar pattern (Figure A2), and the sunflower spiral pattern (Figure A3). In this context, “concentric” describes pivots with the same centre point. Each design was intended to capture the spatial variations of the tear film while relying on a limited number of discrete points. For every pattern, different numbers of pivots were tested, and a feedforward neural network was subsequently trained and validated for each case. Model performance was automatically assessed using a fully automated custom-built MATLAB-coded search function (MathWorks Inc., Natick, MA, USA), with the key metric being the lowest validation error.

Among the configurations tested, the concentric polar pattern proved the most effective, with 121 pivot points giving the highest model accuracy. This number was chosen because it was sufficient to capture meaningful patterns, while larger data sets made the model slower and less efficient. Plotting all 121 points in this section’s figures leaves limited labelling space, leading to overlapping text and reduced clarity. For this reason, to enhance readability in this appendix, the figures are shown with 61 pivots for the concentric ring, 61 for the concentric polar pattern, and 60 for the sunflower spiral pattern instead of the full 121.

An additional consideration was the arrangement of pivots between the right and left eyes. The pivot positions were carefully ordered in a mirrored fashion, ensuring symmetry across the two eyes. This mirroring approach was visually intuitive and demonstrated a measurable improvement in model performance, highlighting the importance of spatial organisation when constructing AI-based predictive frameworks.
